# Shorter‐term and longer‐term mortality prediction in the Australian Diabetes, Obesity and Lifestyle (AusDiab) study

**DOI:** 10.1111/imj.70015

**Published:** 2025-03-25

**Authors:** Dunya Tomic, Agus Salim, Elizabeth L. M. Barr, Paul Z. Zimmet, Dianna J. Magliano, Jonathan E. Shaw

**Affiliations:** ^1^ School of Public Health and Preventive Medicine Monash University Melbourne Victoria Australia; ^2^ Diabetes and Population Health Baker Heart and Diabetes Institute Melbourne Victoria Australia; ^3^ Melbourne School of Population and Global Health The University of Melbourne Melbourne Victoria Australia; ^4^ School of Mathematics and Statistics The University of Melbourne Melbourne Victoria Australia; ^5^ Menzies School of Health Research Charles Darwin University Darwin Northern Territory Australia; ^6^ Department of Diabetes Monash University Melbourne Victoria Australia

**Keywords:** mortality, risk factor, cohort study, cardiovascular disease, diabetes mellitus

## Abstract

**Background:**

While identification of key risk factors for mortality has contributed to advances in healthcare, the effect of these risk factors in predicting mortality over different time horizons remains unclear.

**Aims:**

We sought to determine how risk factors predicted shorter‐term and longer‐term mortality across the age spectrum in adults.

**Methods:**

We used data from 11 247 adults of the Australian Diabetes, Obesity and Lifestyle (AusDiab) study. Cox multivariable regression models were used to estimate hazard ratios (HRs) of shorter‐term (0–10 years) and longer‐term (10–20 years) all‐cause and cardiovascular disease (CVD)‐related mortality associated with risk factors. Models with interaction between baseline age and each risk factor were also fitted.

**Results:**

During a 20‐year follow‐up, 2185 deaths occurred. Smoking, diabetes, male sex and albuminuria all independently predicted shorter‐ and longer‐term all‐cause and CVD mortality. Most associations were stronger in the shorter term compared to the longer term. A notable exception was the association between smoking and CVD mortality, which was stronger in the longer term (HR 3.55, 95% confidence interval (CI) 2.57–4.90) compared to the shorter term (HR 2.06, 95% CI 1.33–3.20). The magnitude of association between most risk factors and mortality attenuated with age.

**Conclusions:**

Classical risk factors for total and CVD mortality remain important up to 20 years after their measurement. In unselected adult cohorts, longer‐term follow‐up (e.g. beyond 10 years) may not provide additional information on associations of risk factors with mortality beyond that obtained in shorter‐term follow‐up.

## Introduction

Identification and management of risk factors for all‐cause and cardiovascular disease (CVD) mortality have enabled great advances in healthcare. Male sex, smoking, diabetes, obesity, hypertension and physical inactivity are among the strongest risk factors for both all‐cause and CVD mortality as identified by numerous population‐based studies.^1^, ^2^, ^3^, ^4^, ^5^, ^6^, ^7^, ^8^ However, the effect of these risk factors in predicting shorter‐term compared to longer‐term mortality remains unclear. It is important to characterise longer‐term mortality risk associated with key factors to understand the public health burden and need for intervention,^9^ yet most studies investigating the effects of risk factors in predicting mortality are limited by relatively short follow‐up periods of 10 years or less.^3^, ^4^, ^10^, ^11^, ^12^ It is also difficult to directly compare data about risk factors from many of these heterogenous studies with varying lengths of follow‐up. As such, we sought to determine how major risk factors predict shorter‐term and longer‐term mortality across the age spectrum in adults, using data from the well‐characterised Australian Diabetes, Obesity and Lifestyle (AusDiab) study.

## Methods

### Study design and population

AusDiab is a national, Australian, population‐based prospective cohort study whose detailed methods have been previously described.^13^ In brief, baseline data were collected from 1999 to 2000 on 11 247 community‐dwelling adults aged 25 years and older, who were recruited from 42 randomly selected census districts across Australia (overall response rate 37%). The baseline survey consisted of an initial household interview followed by a biomedical examination. Two rounds of follow‐up occurred, in 2004–2005 (phase 2) and 2011–2012 (phase 3). A total of 6242 participants (55% of the total cohort) completed both phase 2 and phase 3 examinations. All participants provided informed consent. Ethics approval was granted by the International Diabetes Institute, Monash University and the Australian Institute of Health and Welfare.

### Risk factor measurement

At each examination, data on age, sex, use of antihypertensive and lipid‐lowering medications and smoking (never, ex‐, or current smoker) were collected using questionnaires. Measurements included blood pressure,^14^ anthropometrics,^15^ a fasting blood sample and urine sample. All participants, except those on pharmacotherapy for diabetes, underwent a 75‐g oral glucose tolerance test. Fasting plasma glucose (FPG), 2‐h plasma glucose (2‐h PG), triglycerides and high‐density lipoprotein (HDL) cholesterol were measured with an Olympus AU600 analyser (Olympus Optical, Tokyo, Japan). For exercise status, sufficient exercise was defined as ≥150 min per week, insufficient exercise was defined as 75–149 min/week, and sedentary was defined as <75 min/week according to Active Australia guidelines.^16^ Hypertension was defined as blood pressure ≥140/90 mmHg or receiving antihypertensive medication. Albuminuria, which included both microalbuminuria and macroalbuminuria, was defined as urine albumin to creatinine ratio (ACR) ≥2.5 mg/mmol for men or ≥3.5 mg/mmol for women. Categories of abnormal glucose metabolism were determined according to 1999 World Health Organisation criteria.^17^ Participants were classified into one of the following four categories: known diabetes, new diabetes, impaired fasting glucose (IFG), impaired glucose tolerance (IGT) or normal glucose tolerance (NGT) (Table S1).

### Mortality follow‐up and outcomes

Follow‐up for all‐cause mortality was to 18 June 2019. Cause of death information was only available up to 17 December 2017, so the period of follow‐up for CVD mortality was until this date. Mortality status and underlying cause of death were determined by linking the AusDiab cohort to the National Death Index (NDI) using probabilistic methods based on name, date of birth and sex. Participants not matched to the NDI were assumed to be alive. CVD deaths were defined as those with underlying cause of death codes I10–I25, I46.1, I48, I50–I99 or R96, according to the International Classification of Diseases, Tenth Revision (ICD‐10). Additionally, those with underlying cause of death codes for uncomplicated diabetes mellitus (ICD‐10 codes E109, E119 or E149) or unspecified hyperlipidaemia (E785) were attributed a CVD death if any of the CVD codes were recorded in the first position on the death certificate. For all‐cause mortality, the outcome of interest for shorter‐term mortality was death occurring within 10 years of an individual's baseline examination, while for longer‐term mortality, the outcome was death occurring between 10 and 20 years after baseline examination. For CVD mortality, as only an 18‐year follow‐up period was available, the outcome of interest for shorter‐term mortality was death within 9 years of baseline examination, while for longer‐term mortality it was death occurring between 9 and 18 years after baseline examination.

### Statistical analysis

Cox multivariable regression models were used to estimate hazard ratios (HRs) and 95% confidence intervals (CIs) of all‐cause and CVD mortality associated with risk factors measured at baseline. All risk factors were entered simultaneously into the models. The risk factors included in the models were as follows: male sex (compared to female sex); known diabetes, new diabetes, IFG and IGT (compared to NGT); current and ex‐smoking history (compared to no smoking history); sedentary and insufficient exercise status (compared to sufficient exercise status); hypertension (compared to no hypertension); waist circumference; HDL cholesterol; triglycerides; and albuminuria (compared to no albuminuria). Age was the time scale in the Cox model.

#### Shorter‐term modelling

For shorter‐term mortality, the time at entry was the date of the baseline examination, and the exit time was the date of death or 10 years after baseline examination (for all‐cause mortality) or 9 years after baseline examination (for CVD mortality). The entry age for these models was age at baseline examination.

#### Longer‐term modelling

The analyses of longer‐term mortality excluded any individuals who died within the first 10 years for all‐cause mortality or 9 years for CVD mortality. To avoid immortal time bias,^18^ the follow‐up period started at 10 years after baseline examination (for all‐cause mortality) or 9 years after baseline examination (for CVD mortality). The exit time was 20 years after baseline examination (for all‐cause mortality) or 18 years after baseline examination (for CVD mortality), or date of death if it occurred before this time. The entry age for these longer‐term models was the age at the start of the follow‐up period as defined earlier.

The average age during follow‐up in the cohort used to fit the longer‐term models was older than that of the cohort used to fit the shorter‐term predictive models. As such, an observed difference in the HRs of the two models could simply be due to age‐varying effect size (e.g. a risk factor has a smaller effect size for an older cohort). To examine how much of the difference could be attributed to differences in age profiles between the two cohorts, we fitted Cox proportional hazards models with an interaction term between baseline age and a risk factor. In these models, the log HR was both a function of the risk factor level and baseline age (*A*) through the following model: log HR(*X*,*A* = *a*) = *X*(*β* + (*a* − *μ*_*a*_)*γ*), where *μ*_*a*_ is the average baseline age. The parameter *β* is the log HR for risk factor *X* for those with average baseline age, while for those with baseline age = *a*, the log HR is *β* + (*a* − *μ*_*a*_)*γ*. The *γ* parameter controls the trend in log HR across age; when *γ* > 0, HR increases with age, and the reverse is true when *γ* < 0. Standard errors of HR for a specific age were calculated using the delta method. Separate models were fitted for each risk factor. A similar HR estimate across the age spectrum for both shorter‐ and longer‐term models was taken as evidence that the differences in the overall HR were unlikely to be due to age‐varying effect size.

#### Overall mortality analysis

For both all‐cause and CVD mortality, overall mortality from baseline until the end of the follow‐up period associated with various risk factors was also analysed. In this analysis, the time at entry was the date of baseline examination and exit time was 20 years after baseline examination (for all‐cause mortality), 18 years after baseline examination (for CVD mortality) or date of death if earlier.

#### Subgroup analysis

A subgroup analysis of the individuals who underwent subsequent examinations in 2004–2005 (phase 2) and 2011–2012 (phase 3) was conducted to determine whether the association between risk factors and longer‐term all‐cause mortality was stronger using data from follow‐up visits compared to baseline data. In this analysis, HR and 95% CI of 12‐ to 20‐year all‐cause mortality associated with the risk factors were estimated in three separate models. The first model used baseline measurements, the second used measurements taken at phase 2 re‐examination in 2004–2005, and the third used measurements taken at phase 3 re‐examination in 2011–2012.

Stata, version 17.0 (College Station, TX, USA) was used for all analyses.

## Results

### Baseline characteristics

This study included 11 247 AusDiab participants in the analysis of all‐cause mortality (Table 1), 11 220 of whom were included in the analysis of CVD mortality. As 27 participants were matched to the NDI but had no cause of death data available, these participants were excluded from the cause‐specific analysis. A total of 3578 individuals had follow‐up testing data from both 2004–2005 and 2011–2012 for all variables included in this analysis. The overall cohort had a median baseline age of 50 (interquartile range 40–62) and was 55.1% female. Almost three‐quarters (73.2%) had normal glucose tolerance. Approximately half (52.1%) of the cohort undertook sufficient exercise to meet guidelines, and 55.0% had never smoked. Around two‐thirds (67.3%) were normotensive, and most (92.6%) had no albuminuria. The baseline characteristics of those with follow‐up visits were mostly aligned to those of the overall cohort. However, those who underwent follow‐up visits were less likely to be current smokers (9.8% vs 15.8%) compared to the overall cohort.

**Table 1:** Baseline characteristics of AusDiab study population included in analysis of all‐cause mortality (*n* = 11 247) and subgroup analysis of cohort undergoing follow‐up testing (*n* = 3578)

	All‐cause mortality population (*n* = 11 247)	Follow‐up testing population (*n* = 3578)
Age (years), median (IQR)	50 (40–62)	49 (41–57)
Sex, *n* (%)		
Women	6199 (55.1)	1993 (55.7)
Men	5048 (44.9)	1585 (44.3)
Diabetes status, *n* (%)		
Known diabetes	475 (4.3)	84 (2.4)
New diabetes	466 (4.2)	100 (2.8)
Impaired fasting glucose	650 (5.9)	199 (5.6)
Impaired glucose tolerance	1378 (12.4)	334 (9.4)
Normal glucose tolerance	8107 (73.2)	2825 (79.8)
Smoking status, *n* (%)		
Current smoker	1745 (15.8)	348 (9.8)
Ex‐smoker	3218 (29.2)	910 (25.7)
Non‐smoker	6072 (55.0)	2282 (64.5)
Exercise status, *n* (%)		
Sedentary	1920 (17.2)	535 (15.0)
Insufficient	3424 (30.7)	1107 (31.1)
Sufficient	5817 (52.1)	1923 (53.9)
Hypertension, *n* (%)		
Yes	3649 (32.7)	918 (25.7)
No	7525 (67.3)	2650 (74.3)
Waist circumference (cm), median (IQR)	90.5 (80.8–100.0)	89.0 (79.4–98.3)
HDL cholesterol (mmol/L), median (IQR)	1.4 (1.2–1.7)	1.4 (1.2–1.7)
Triglycerides (mmol/L), median (IQR)	1.3 (0.9–1.9)	1.2 (0.8–1.8)
Albuminuria, *n* (%)		
Yes	833 (7.5)	134 (3.8)
No	10 349 (92.6)	3435 (96.3)

Sufficient exercise was defined as ≥150 min/week of physical activity, insufficient exercise was defined as 75–149 min/week, and sedentary was defined as <75 min/week. Hypertension was defined as blood pressure ≥140/90 mmHg or receiving antihypertensive medication. Albuminuria, which included both microalbuminuria and macroalbuminuria, was defined as urine albumin to creatinine ratio (ACR) ≥2.5 mg/mmol for men or ≥3.5 mg/mmol for women.

CVD, cardiovascular disease; HDL, high‐density lipoprotein; IQR, interquartile range.

### All‐cause mortality

A total of 2185 deaths occurred over the 20‐year follow‐up. Of these deaths, 855 occurred within the first 10 years of follow‐up, while 1330 occurred between 10 and 20 years of follow‐up. Male sex, known diabetes, current smoking status and albuminuria were all associated with both shorter‐term and longer‐term all‐cause mortality (Table 2). Current smoking status was the strongest predictor of both shorter‐term and longer‐term mortality. The multivariable adjusted HR for all‐cause mortality associated with current smoking status was similar in magnitude in the shorter‐term (HR 2.61, 95% CI 2.13–3.20) and the longer‐term (HR 2.32, 95% CI 1.96–2.74). The association between sex, known diabetes and albuminuria and all‐cause mortality was stronger for shorter‐term mortality compared to longer‐term mortality.

**Table 2:** Association between risk factors and shorter‐term (0–10 years) and longer‐term (10–20 years) all‐cause mortality in the AusDiab study cohort (*n* = 11 247)

	Mortality 0–10 years	Mortality 10–20 years	Mortality 0–20 years
Sex			
Female	0.68 (0.57–0.81)	0.80 (0.70–0.92)	0.75 (0.68–0.84)
Diabetes status			
Known diabetes	1.64 (1.31–2.04)	1.47 (1.20–1.78)	1.54 (1.33–1.78)
New diabetes	1.39 (1.09–1.76)	1.01 (0.81–1.25)	1.16 (0.99–1.36)
Impaired fasting glucose	1.32 (1.00–1.75)	1.05 (0.83–1.31)	1.14 (0.96–1.36)
Impaired glucose tolerance	1.31 (1.10–1.57)	1.15 (0.99–1.33)	1.20 (1.07–1.35)
Smoking status			
Current smoker	2.61 (2.13–3.20)	2.32 (1.96–2.74)	2.42 (2.13–2.76)
Ex‐smoker	1.31 (1.12–1.53)	1.08 (0.95–1.22)	1.17 (1.06–1.29)
Exercise status			
Sedentary	1.39 (1.17–1.66)	1.07 (0.92–1.24)	1.17 (1.04–1.31)
Insufficient	1.13 (0.96–1.33)	1.02 (0.90–1.16)	1.06 (0.96–1.17)
Hypertension	1.11 (0.94–1.30)	1.21 (1.07–1.37)	1.16 (1.05–1.28)
Waist circumference (cm)	1.00 (0.99–1.01)	1.01 (1.01–1.02)	1.01 (1.00–1.01)
HDL cholesterol (mmol/L)	1.10 (0.88–1.38)	0.97 (0.80–1.17)	1.02 (0.89–1.17)
Triglycerides (log value) (mmol/L)	0.98 (0.83–1.15)	1.01 (0.89–1.15)	1.01 (0.91–1.11)
Albuminuria	1.51 (1.28–1.79)	1.31 (1.11–1.54)	1.37 (1.22–1.54)

Sufficient exercise was defined as ≥150 min/week of physical activity, insufficient exercise was defined as 75–149 min/week, and sedentary was defined as <75 min/week. Hypertension was defined as blood pressure ≥140/90 mmHg or receiving antihypertensive medication. Albuminuria, which included both microalbuminuria and macroalbuminuria, was defined as urine albumin to creatinine ratio (ACR) ≥2.5 mg/mmol for men or ≥3.5 mg/mmol for women. All reported HR values are adjusted for all covariates in the table.

CI, confidence intervals; HDL, high‐density lipoprotein; HR, hazard ratio.

**Figure 1:**
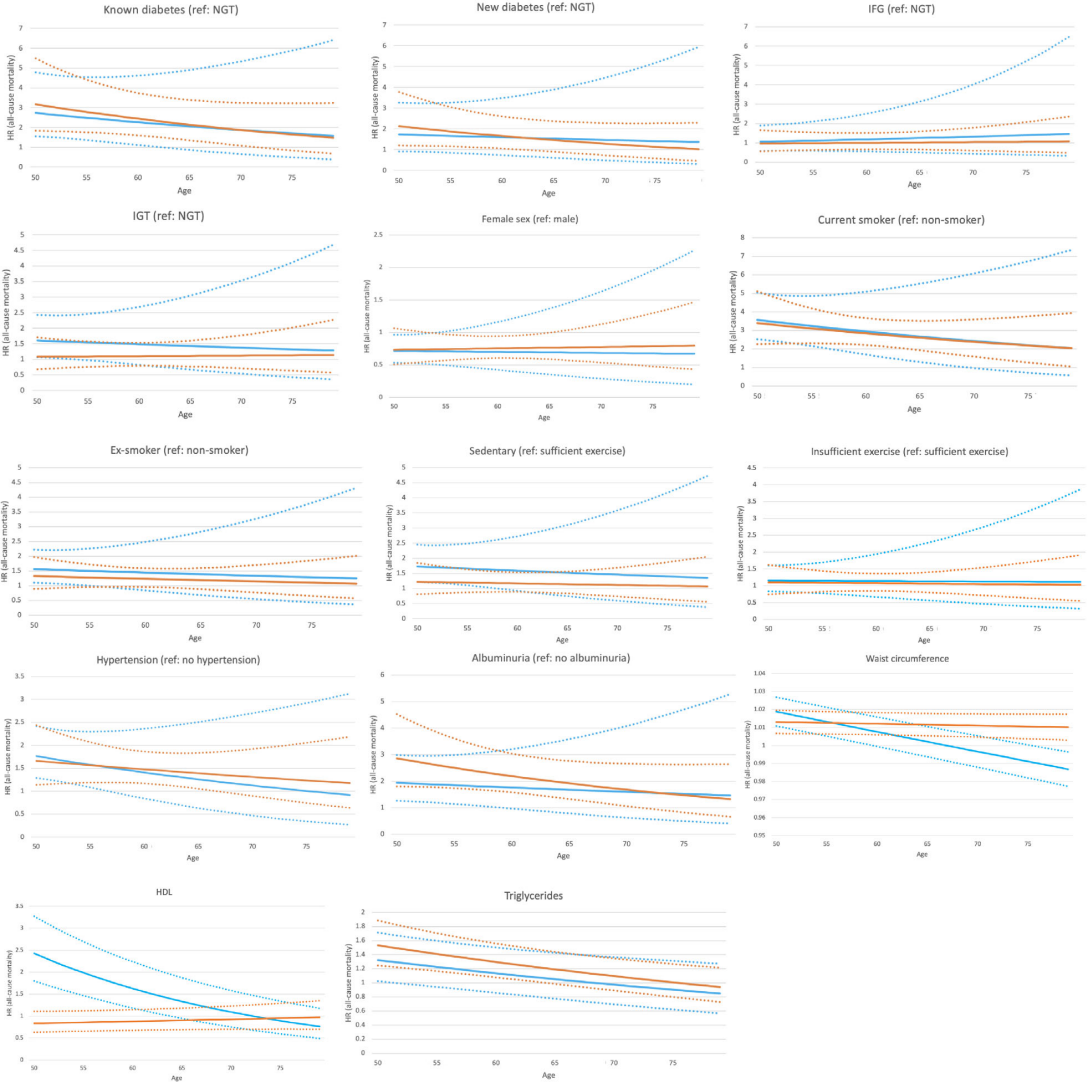
Association between risk factors and shorter‐term (0–10 years) and longer‐term (10–20 years) all‐cause mortality across the age spectrum in the AusDiab study cohort (*n* = 11 247) (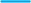) Short‐term mortality (0‐10 years) (point estimate), (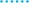) Short‐term mortality (0‐10 years) (95% CI), (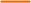) Long‐term mortality (10‐20 years) (point estimate), (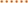) Long‐term mortality (10‐20 years) (95% CI). CI, confidence interval; HDL, high‐density lipoprotein; HR, hazard ratio; IFG, impaired fasting glucose; IGT, impaired glucose tolerance; NGT, normal glucose tolerance. Sufficient exercise was defined as ≥150 min/week of physical activity, insufficient exercise was defined as 75–149 min/week, and sedentary was defined as <75 min/week. Hypertension was defined as blood pressure ≥140/90 mmHg or receiving antihypertensive medication. Albuminuria, which included both microalbuminuria and macroalbuminuria, was defined as urine albumin to creatinine ratio (ACR) ≥2.5 mg/mmol for men or ≥3.5 mg/mmol for women.

Figure 1 shows that the associations of most risk factors with both shorter‐ and longer‐term mortality weakened slightly with age. Furthermore, Figure 1 demonstrates that the influence of follow‐up time on these associations was significantly attenuated when considering the risk at any given age.

The magnitude of association between risk factors and 0‐ to 20‐year all‐cause mortality was generally smaller than that observed for 0‐ to 10‐year mortality but greater than for 10‐ to 20‐year mortality. Current smoking status was the strongest predictor of 0‐ to 20‐year all‐cause mortality (HR 2.42, 95% CI 2.13–2.76).

### Cardiovascular disease mortality

Among those included in the cause‐specific mortality follow‐up (*n* = 11 220), 576 CVD deaths occurred over 18 years of follow‐up. Of these deaths, 236 occurred in the first 9 years, while 340 occurred between 9 and 18 years of follow‐up. As for all‐cause mortality, male sex, known diabetes, current smoking status and albuminuria were all associated with both shorter‐term and longer‐term CVD mortality in multivariable models (Table 3). Current smoking status was the strongest predictor of CVD mortality. This association was much stronger in the longer term (HR 3.55, 95% CI 2.57–4.90) compared to the shorter term (HR 2.06, 95% CI 1.33–3.20). The associations of sex and known diabetes were stronger for shorter‐term compared to longer‐term CVD mortality, while the association between albuminuria and shorter‐term CVD mortality was marginally weaker compared to that for longer‐term mortality. Except for current smoking status, the magnitude of association between all risk factors and CVD mortality was largely aligned to that for all‐cause mortality. As with all‐cause mortality, the magnitude of association between risk factors and overall, 0‐ to 18‐year CVD mortality was generally intermediate between that of 0‐ to 9‐year and of 9‐ to 18‐year mortality, with the strongest association observed for current smoking status (HR 2.96, 95% CI 2.29–3.82).

**Table 3:** Association between risk factors and shorter‐term (0–9 years) and longer‐term (9–18 years) cardiovascular disease mortality among individuals in the AusDiab study cohort with cause of death information (*n* = 11 220)

	CVD mortality 0–9 years	CVD mortality 9–18 years	CVD mortality 0–18 years
Sex			
Female	0.67 (0.48–0.93)	0.77 (0.59–1.02)	0.70 (0.57–0.87)
Diabetes status			
Known diabetes	1.56 (1.05–2.32)	1.50 (1.05–2.14)	1.62 (1.24–2.10)
New diabetes	1.29 (0.82–2.00)	0.98 (0.66–1.47)	1.12 (0.83–1.50)
Impaired fasting glucose	1.75 (1.06–2.87)	0.89 (0.55–1.46)	1.20 (0.85–1.68)
Impaired glucose tolerance	1.17 (0.83–1.66)	0.98 (0.73–1.32)	1.07 (0.86–1.34)
Smoking status			
Current smoker	2.06 (1.33–3.20)	3.55 (2.57–4.90)	2.96 (2.29–3.82)
Ex‐smoker	1.32 (0.99–1.76)	1.14 (0.89–1.47)	1.25 (1.03–1.51)
Exercise status			
Sedentary	1.16 (0.81–1.65)	1.14 (0.85–1.53)	1.16 (0.93–1.46)
Insufficient	1.52 (1.13–2.04)	1.10 (0.86–1.41)	1.26 (1.04–1.52)
Hypertension	1.37 (0.98–1.91)	1.28 (0.99–1.66)	1.32 (1.08–1.61)
Waist circumference (cm)	1.00 (0.99–1.02)	1.01 (1.00–1.02)	1.01 (1.00–1.02)
HDL cholesterol (mmol/L)	0.74 (0.47–1.15)	0.96 (0.65–1.40)	0.85 (0.64–1.14)
Triglycerides (log value) (mmol/L)	0.77 (0.56–1.06)	0.97 (0.75–1.27)	0.87 (0.71–1.06)
Albuminuria	1.48 (1.09–2.01)	1.50 (1.12–2.02)	1.55 (1.26–1.90)

Sufficient exercise was defined as ≥150 min/week of physical activity, insufficient exercise was defined as 75–149 min/week, and sedentary was defined as <75 min/week. Hypertension was defined as blood pressure ≥140/90 mmHg or receiving antihypertensive medication. Albuminuria, which included both microalbuminuria and macroalbuminuria, was defined as urine albumin to creatinine ratio (ACR) ≥2.5 mg/mmol for men or ≥3.5 mg/mmol for women. All reported HR values are adjusted for all covariates in the table.

CI, confidence intervals; CVD, cardiovascular disease; HDL, high‐density lipoprotein; HR, hazard ratio.

### Cohort with repeated risk factor measurements

Among AusDiab participants who attended both phase 2 and phase 3 visits (*n* = 3578), 226 deaths occurred after the phase 3 visit at 12 years. Current smoking status was the only predictor of longer‐term all‐cause mortality in this cohort using data on risk factor measurements at each phase of the study (Table 4). The association between smoking and all‐cause mortality was strongest using the most recent (2011–2012) phase 3 data (HR 3.90, 95% CI 2.34–6.53) and weakest using baseline data (HR 2.90, 95% CI 1.88–4.47).

**Table 4:** Associations of risk factors, at baseline, phase 2 and phase 3 examinations, with 12‐ to 20‐year all‐cause mortality among individuals who underwent follow‐up testing in the AusDiab study (*n* = 3578)

	Mortality 12–20 years (using baseline data from 1999 to 2000)	Mortality 12–20 years (using phase 2 data from 2004 to 2005)	Mortality 12–20 years (using phase 3 data from 2011 to 2012)
Sex			
Female	0.76 (0.54–1.09)	0.84 (0.59–1.21)	0.72 (0.51–1.01)
Diabetes status			
Known diabetes	1.62 (0.93–2.84)	1.30 (0.81–2.10)	1.69 (1.13–2.53)
New diabetes	1.61 (0.94–2.77)	1.12 (0.59–2.13)	1.03 (0.49–2.14)
Impaired fasting glucose	1.54 (0.95–2.50)	0.94 (0.50–1.76)	1.23 (0.67–2.23)
Impaired glucose tolerance	1.04 (0.70–1.53)	1.22 (0.83–1.80)	0.93 (0.63–1.36)
Smoking status			
Current smoker	2.90 (1.88–4.47)	3.04 (1.90–4.87)	3.90 (2.34–6.53)
Ex‐smoker	0.88 (0.64–1.22)	1.08 (0.79–1.46)	1.09 (0.81–1.47)
Exercise status			
Sedentary	1.18 (0.80–1.74)	1.01 (0.66–1.52)	1.18 (0.82–1.70)
Insufficient	0.87 (0.63–1.19)	1.10 (0.82–1.48)	1.05 (0.76–1.45)
Hypertension	1.20 (0.88–1.63)	1.26 (0.93–1.71)	0.86 (0.64–1.17)
Waist circumference (cm)	1.01 (0.99–1.02)	1.01 (1.00–1.03)	1.00 (0.99–1.02)
HDL cholesterol (mmol/L)	0.87 (0.55–1.40)	1.07 (0.69–1.65)	0.75 (0.81–1.12)
Triglycerides (log value) (mmol/L)	0.88 (0.63–1.22)	1.03 (0.72–1.49)	0.91 (0.64–1.29)
Albuminuria	1.33 (0.81–2.18)	1.09 (0.72–1.67)	1.40 (1.02–1.92)

Baseline risk factor measurements were taken in financial year 1999/2000, while 5‐year and 12‐year risk factor measurements were taken in financial years 2004/2005 and 2011/2012 respectively. Sufficient exercise was defined as ≥150 min/week of physical activity, insufficient exercise was defined as 75–149 min/week, and sedentary was defined as <75 min/week. Hypertension was defined as blood pressure ≥140/90 mmHg or receiving antihypertensive medication. Albuminuria, which included both microalbuminuria and macroalbuminuria, was defined as urine albumin to creatinine ratio (ACR) ≥2.5 mg/mmol for men or ≥ 3.5 mg/mmol for women. All reported HR values are adjusted for all covariates in the table.

CI, confidence intervals; HDL, high‐density lipoprotein; HR, hazard ratio.

## Discussion

In this large, national population‐based prospective cohort of 11 247 adults, most risk factors predicted shorter‐term mortality with a larger magnitude than for longer‐term mortality. Current smoking status was the strongest predictor of shorter‐term and longer‐term all‐cause and CVD mortality. The associations between most risk factors and both shorter‐term and longer‐term all‐cause mortality weakened with age. This is the first study to report the effects of a range of risk factors on both all‐cause and CVD mortality in the shorter and longer term in a population‐based cohort.

Our findings of current smoking status and known diabetes as the strongest predictors of mortality are consistent with results from a previous study by Yusuf *et al*.,^3^ which investigated the effect of modifiable risk factors on mortality and major CVD events in 155 722 adults from 21 countries. Over a median follow‐up time of 9.5 years, which aligns with our 10‐year follow‐up period for shorter‐term mortality, current smoking status was the strongest predictor of mortality (HR 1.74, 95% CI 1.61–1.88). The authors also found diabetes to be a strong predictor of major CVD, defined as CVD death, myocardial infarction, stroke and heart failure (HR 1.74, 95% CI 1.61–1.88). The study by Pencina *et al*. using Framingham Heart Study data^19^ found that male sex (HR 1.73, 95% CI 1.45–2.07), smoking (HR 2.01, 95% CI 1.72–2.35), diabetes (HR 2.49, 95% CI 1.82–3.41), systolic blood pressure (HR 1.29, 95% CI 1.19–1.39) and HDL cholesterol (HR 0.78, 95% CI 0.72–0.84) were all associated with 30‐year CVD events. We also similarly found that male sex, smoking, hypertension and diabetes were significantly associated with longer‐term CVD mortality, albeit over a shorter follow‐up duration of 18 years. However, in our study, HDL‐cholesterol was not significantly associated with CVD mortality. HDL cholesterol has been considered for many years to be inversely associated with CVD risk.^20^ A recent study of 11 987 people with hypertension found a U‐shaped association between HDL levels and the risk of CVD events in males. However, there was no increased CVD risk associated with elevated HDL levels in females.^21^ Furthermore, Mendelian randomisation studies do not support a causal role of HDL cholesterol in CVD events.^22^ In this context, our results for HDL are not surprising.

Waist circumference did not emerge as a risk factor for either all‐cause or CVD mortality, although the odds ratios were borderline, so an association cannot be excluded. Findings from other cohort studies of waist circumference and mortality risk are inconsistent,^23^, ^24^, ^25^, ^26^ reflecting its absence from most CVD risk calculators.^27^ We presented data adjusted for diabetes status, hypertension and lipids, which often mediate the effects of adiposity.^28^, ^29^ Although prone to measurement error,^30^ waist circumference can be a useful clinical tool for assessing adiposity and for estimating risks for more closely related outcomes such as diabetes. Similarly, we found no association between triglycerides and mortality. The relationship between triglycerides and mortality is often attenuated after adjusting for other CVD risk factors,^31^ and it remains unclear whether triglyceride concentration is independently related to CVD or all‐cause mortality.^32^ As such, triglycerides are also absent from most CVD risk calculators.^33^

We found that the relationship between many risk factors and mortality diminished with increasing age. This likely explains the larger effect sizes observed for shorter‐term mortality compared to longer‐term mortality in some instances, since the shorter follow‐up time, by design, includes events at a younger age than does longer follow‐up. There are two main reasons that risk prediction may weaken over time. First, the risk status may change over time, and so the baseline assessment becomes less relevant. Second, the relative effect of the risk factor may diminish as people age. Our observation that the risk associated with sex diminished at longer follow‐up supports the second of these reasons, as sex, unlike other risk factors, does not change with time. The nature of interactions between risk factors and age will be influenced by many simultaneously occurring processes, some of them offsetting the impact of others. Therefore, it is not surprising that studies investigating the effects of risk factors according to age have produced conflicting results. Several studies observed declines in the effects of risk factors including education, socioeconomic status and ethnicity with age, with most citing mortality selection as a key explanation for these findings.^34^, ^35^, ^36^

Longer‐term follow‐up beyond 10 years is often essential in cohort studies for sufficient numbers of participants to develop outcomes so that risk factors can be comprehensively explored.^37^ However, our results suggest that this may not be necessary in studies of CVD risk factors where mortality is the outcome and the population is composed of mostly middle‐aged or older adults, as was the case in our study which had a median age of 50 years. Researchers of similar studies of CVD risk factors should consider whether the additional information gained by extending follow‐up periods is likely to justify the increased costs and time commitments for participants.^38^ It is unclear whether the utility of additional follow‐up beyond 10 years varies according to the nature of the risk being studied, and so similar analyses in other cohort studies, for example, those investigating cancer risk, may be useful.

The major strengths of this study include the large, population‐based prospective cohort design and the collection of data for a wide range of variables that we were able to analyse as risk factors in our analysis. One of the limitations is the response rate of only 37% of those eligible for testing at baseline, indicating that the study cohort may not be fully representative of the Australian adult population. The study relied on results from a single measurement at each visit to determine participants’ risk factor status, so misclassification may have occurred.^39^ This may in part explain the lack of observed associations for some of the risk factors that show most variability, such as blood pressure,^40^ while the most significant associations were observed for the more consistent variables like sex and smoking status. Less than one‐third of the cohort had repeated risk factor measurements, meaning that the precision of all estimates was decreased in the subgroup analysis, despite stable effect sizes compared to those reported in the overall cohort. As such, only smoking status, which was the strongest predictor of longer‐term mortality in all analyses and one of the least prone to misclassification, remained an independent predictor of longer‐term mortality in this group.

## Conclusion

In summary, with results from this large, population‐based cohort study, we provide information to clinicians and public health decision‐makers about the effects of risk factors in predicting shorter‐term and longer‐term all‐cause and CVD mortality. As most associations of risk factors with mortality were stronger in the shorter term, our findings suggest that researchers should consider the necessity of longer‐term follow‐up in similar future cohort studies of CVD risk factors.

## Supporting information


**Data S1.** Supporting Information.
