# Supplementary material for: Shorter‐term and longer‐term mortality prediction in the Australian Diabetes, Obesity and Lifestyle (AusDiab) study
Source: Intern Med J. 2025 Mar 25;55(6):922–31. doi: 10.1111/imj.70015 (PMC12155081; doi:10.1111/imj.70015)
Supplement: Supplementary file 1 — Data S1. Supporting Information. [file IMJ-55-922-s001.docx]

**Supporting information**

Supplementary Table 1: Classification of AusDiab participants into glucose metabolism categories

| **Category** | **Criteria** |
| --- | --- |
| Known diabetes | Diagnosed with diabetes by a physician and either receiving glucose-lowering medication or FPG ≥7.0 mmol/L or 2-hour PG ≥11.1 mmol/L |
| New diabetes | Did not report having diabetes but had FPG ≥7.0 mmol/L or 2-hour PG ≥11.1 mmol/L |
| Impaired fasting glucose | FPG ≥6.1 and <7.0 mmol/L; 2-hour PG <7.8 mmol/L |
| Impaired glucose tolerance | 2-hour PG ≥7.8 and <11.1 mmol/L; FPG <7.0 mmol/L |
| Normal glucose tolerance | FPG <6.1 mmol/L; 2-hour PG <7.8 mmol/L |

*FPG = fasting plasma glucose; 2-hour PG = 2-hour plasma glucose.*
